# Performance and Fouling Study of Asymmetric PVDF Membrane Applied in the Concentration of Organic Fertilizer by Direct Contact Membrane Distillation (DCMD)

**DOI:** 10.3390/membranes8010009

**Published:** 2018-02-16

**Authors:** Yanfei Liu, Tonghu Xiao, Chenghuan Bao, Jifei Zhang, Xing Yang

**Affiliations:** 1 Faculty of Materials Science and Chemical Engineering, Ningbo University, Ningbo 315211, China; liuyanfei2716@163.com (Y.L.); 17855828741@163.com (C.B.); zhangjifei_nbu@163.com (J.Z.); 2 College of Engineering and Science, Victoria University, P.O. Box 14428, Melbourne, VIC 8001, Australia

**Keywords:** direct contact membrane distillation, asymmetric PVDF membrane, concentration of organic fertilizer, anti-fouling

## Abstract

This study proposes using membrane distillation (MD) as an alternative to the conventional multi-stage flushing (MSF) process to concentrate a semi-product of organic fertilizer. By applying a unique asymmetric polyvinylidene fluoride (PVDF) membrane, which was specifically designed for MD applications using a nonsolvent thermally induced phase separation (NTIPS) method, the direct contact membrane distillation (DCMD) performance was investigated in terms of its sustainability in permeation flux, fouling resistance, and anti-wetting properties. It was found that the permeation flux increased with increasing flow rate, while the top-surface facing feed mode was the preferred orientation to achieve 25% higher flux than the bottom-surface facing feed mode. Compared to the commercial polytetrafluoroethylene (PTFE) membrane, the asymmetric PVDF membrane exhibited excellent anti-fouling and sustainable flux, with less than 8% flux decline in a 15 h continuous operation, i.e., flux decreased slightly and was maintained as high as 74 kg·m^−2^·h^−1^ at 70 °C. Meanwhile, the lost flux was easily recovered by clean water rinsing. Overall 2.6 times concentration factor was achieved in 15 h MD operation, with 63.4% water being removed from the fertilizer sample. Further concentration could be achieved to reach the desired industrial standard of 5x concentration factor.

## 1. Introduction

Livestock manure and crop straw have been used as fertilizer as they are rich in nitrogen, phosphorus, and organic matter that can improve the physical and chemical properties of soil and provide nutrients essential to crops [1]. However, such fertilizers need to be concentrated to a certain level, i.e., at least 3–5 times concentration from an initial organic content of 3%, to achieve the desired nutrient strength. Currently, several technologies have been applied in industry to concentrate liquid fertilizers, such as multistage flash distillation (MSF), multiple-effect distillation (MED), or reverse osmosis (RO). Both MSF and MED plants are known to be inefficient, energy-intensive, and land-consuming [2]. The major limitation of RO in such applications is its relatively low water removal (~35%) due to the high osmotic pressure limited by the concentration effect and thus the low overall concentration factor (<1.0) [3,4]. Also, RO is highly susceptible to membrane fouling [5]. 

Membrane distillation (MD) is an alternative emerging technology that combines the comparative advantages of thermal distillation and membrane processes and involves the transport of water vapor across a microporous hydrophobic membrane [6,7]. The driving force of MD is supplied by the vapor pressure difference generated by the temperature gradient imposed between the liquid/vapor interfaces [8]. Compared to other separation processes, MD has many advantages [9]. It exhibits a complete rejection of dissolved, non-volatile species, and lower (ambient) operating pressure than the pressure-driven membrane processes. Highly saturated solutions can be treated in MD [10]. Meanwhile, MD has the potential to achieve a high concentration factor while operating at low temperature differences that are achievable using waste-grade waste heat [11] or a renewable energy source, such as solar and geothermal energy [12,13]. MD has the potential to concentrate and recover valuable resources. For example, MD has been widely investigated for desalination [14], concentration of juices [15], crystallization of minerals [16], recovery of volatiles such as nitrogen [17], and waste water purification [18] and treatment [19] in recent years. However, the concentration of organic fertilizer using MD has not been studied thus far, where abundant waste heat (70–80 °C) will be available from the fertilizer production process [20].

Although MD has great potential for treating highly concentrated solutions, membrane fouling in MD is inevitable in the treatment of real industrial effluents [21]. Fouling results in a decrease in membrane permeability due to a deposition of suspended or dissolved substances, including organic and inorganic components, on the membrane surface and within its pores, reducing the effective vapor transport area and causing potential pore wetting problems that are detrimental to MD performance [22]. In the dewatering process of aqueous solutions such as juice [23] and RO brines [24], the occurrence of fouling on the MD membrane surface is highly possible but this aspect has not been thoroughly investigated [21]. Fouling control in MD lies in the process operating strategies (i.e., hydrodynamics) [25] and membrane properties [26] (i.e., surface roughness and hydrophobicity, etc.). In particular, the development of suitable MD membranes for sustaining the concentration processes is desirable. The long-term stability of the membranes in terms of deterioration of hydrophobicity and pore wetting needs to be resolved. To date, no commercial membranes with superior anti-fouling have been specifically developed for MD applications. Overall, the implementation of MD on an industrial scale is limited by the availability of robust membranes. 

Recent studies showed that most of the MD membranes currently used are fabricated for other processes, such as microfiltration (MF), due to the similar hydrophobic nature and the microporous structure [27]. The desired MD performance with high permeability, long-term stability, and high energy efficiency is typically associated with the following membrane characteristics: a relatively small maximum pore size, the highest possible porosity, a narrow pore size distribution with a high degree of pore interconnectivity, and good anti-wetting properties with high liquid entry pressure of water (LEPw) [28,29]. The membrane properties directly affect the membrane performance and, therefore, an optimized membrane specifically designed for MD is vital for implementing industrial applications [27]. Common membrane materials include poly (vinylidene fluoride) (PVDF), which is widely used for fabricating MD membranes via various fabrication methods, such as conventional nonsolvent induced phase separation (NIPS) [30] and thermally induced phase separation (TIPS) [31], as well as the recently proposed nonsolvent thermally induced phase separation (NTIPS, also referred to as combined NIPS and TIPS) [32]. Our recent work [32] showed that a unique asymmetric PVDF membrane could be fabricated via the NTIPS method to achieve an ultra-thin separation skin layer with a highly porous and interconnected pore structure. Such a membrane exhibited extraordinary permeability as high as 85.6 kg·m^−2^·h^−1^ at 80 °C. 

In this MD study a previously developed polyvinylidene fluoride (PVDF) membrane was applied in the concentration of liquid organic fertilizer. The membrane was prepared by the nonsolvent thermally induced phase separation (NTIPS) method and exhibited superior permeability and anti-wetting properties with a unique asymmetric structure. Firstly, the effects of operating parameters in direct contact membrane distillation (DCMD) were investigated with the as-prepared PVDF membrane, such as flow rate, membrane orientation, and solution salinity. Secondly, the application of the membrane in the dewatering of real organic fertilizer stream to the desired concentration was examined in terms of the process stability and membrane fouling behavior, which was then compared with the commercial polytetrafluoroethylene (PTFE) membrane after previous systematic research into industrial applications [33,34,35].

## 2. Experimental

### 2.1. Membranes

The asymmetric poly(vinylidene fluoride) membranes used in this study were prepared by the nonsolvent thermally induced phase separation (NTIPS) method, with 15 wt % PVDF polymer (Model: 1015, Solvay Co, Brussels, Belgium) dissolved into water-soluble diluent ε-Caprolactam (CPL, Sinopharm Reagent Inc, Shanghai, China) at 150 °C. The nascent membrane was obtained at 20 °C in a coagulation bath with deionized water. Details on the membrane preparation and characterization can be found elsewhere [32]. The commercial polytetrafluoroethylene (PTFE) membrane provided by Ningbo Changqi Porous Membrane Technology Co., Ltd. (Ningbo, China) was also used in this work. 

### 2.2. Feed Solutions

Three synthetic solutions were prepared as feed in MD with various salt concentrations C_f_,: (1) bitter salt solution: 1.7 wt % sodium chloride (NaCl, 99.5%, Sinopharm Reagent Inc, Shanghai, China); (2) synthetic seawater: 3.5 wt % NaCl; (3) 6.0 wt % NaCl solution.

The organic fertilizer sample was obtained from the Environmental Technology Development Co., Ltd. (Ningbo, China). It is a semi-product in the fertilizer production process, made of mixed solution of manure and milled crop straw after purification, pressurized hydrolysis, and pH adjustment. This stream coming from the pressurized hydrolysis process carries certain thermal energy (70 °C) that could be readily used in MD for dewatering. Based on the “Organic Fertilizer Content Standard” (DB33/699-2008) formulated by the National Center for Fertilizer Inspection and Supervision (Beijing, China), the company expected to concentrate the organic matter of the fertilizer product to 15%. The adjusted pH of the sample is within the range of 4.0–8.0 with minimal volatile ammonia nitrogen present. However, the semi-product of the organic fertilizer has only low organic matter around 3%, which needs to be concentrated about 5x to achieve useful strength for industrial applications. Thus, dewatering or concentration of the semi-product will be conducted in MD. 

### 2.3. Membrane Characterization

The top/bottom surface and cross-sections of PVDF flat sheet membrane were observed using a scanning electron microscope (SEM, NOVA NANOSEM 450, FEI, Hillsboro, OR, USA). Prior to the scan, membrane samples were immersed in liquid nitrogen, fractured, and then coated with platinum using a coater (VACUUM DEVICE MSP-1S, FEI, Hillsboro, OR, USA).

The overall membrane porosity (ε) was calculated from the ratio of the pore volume to the total volume of the membrane. The membrane pore volume was determined by measuring the dry and wet weights of membrane using isopropyl alcohol (IPA) as a wetting agent [36]. 

The measurement of liquid entry pressure of water (LEP_w_) of the membranes was conducted using a customized setup with synthetic seawater (i.e., 3.5 wt % NaCl solution, conductivity ~60 ms·cm^−1^) as the testing liquid on the feed side and DI water (conductivity < 10 μs·cm^−1^) as the reference at the permeate side to detect the occurrence of pore wetting. During testing, the pressure of the NaCl solution side was increased steadily using compressed N_2_ gas, by 0.01 MPa increments every 15 min. The pressure at which there was a drastic initial increase in the conductivity of the permeate side and a continuous conductivity increase was taken as the LEP. The conductivity of the solution was monitored by a conductivity meter (DDSJ-308A, INESA Instrument, Shanghai, China).

The mean pore size of the PVDF membrane was determined by the liquid–liquid displacement method based on an isobutanol–DI water system. The detailed experimental procedure can be found elsewhere [37].

The contact angle (CA) of prepared PVDF membranes is measured by a goniometer (Kruss DSA100, Hamburg, Germany). Five points on each membrane are tested and the average of the measured values is reported.

### 2.4. DCMD Experiments

**Figure 1:**
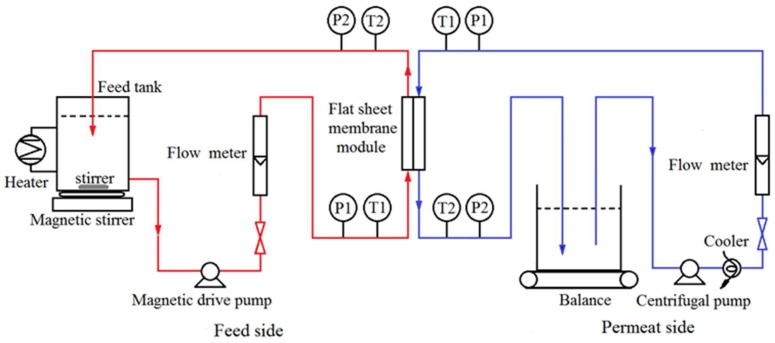
DCMD experimental setup.

The DCMD experiments were conducted with the laboratory setup shown in Figure 1. In all DCMD experiments, the membrane was installed into a flat sheet membrane cell, giving an effective membrane area of 10 × 10^−4^ m^2^. The feed and permeate were flowing counter-currently, with the feed pumped through a magnetic drive pump at a flow rate range of Q_f_ = 50–110 L/h and the permeate recirculated through another centrifugal pump at Q_p_ = 50–110 L/h. A magnetic stirrer was used in the feed tank to improve the mixing of solutions. The feed temperature T_f_ is in the range of 50–80 °C and permeate temperature T_p_ was kept constant at 16 °C. Both synthetic solutions and real industrial samples were tested under the identified operating conditions through this study. The continuous weight gain of the distillate was measured using a digital balance (EK-2000i, A&D Co. Ltd., Tokyo, Japan) for membrane flux calculation. The total dissolved solids (TDS) of the permeate stream was monitored by the conductivity meter to calculate rejection of non-volatiles. For each membrane, DCMD experiments were repeated three times to ensure reproducibility.

### 2.5. Evaluation of DCMD Performance

The permeation flux (J, Kg·m^−2^·h^−1^) in MD was calculated by Equation (1):

(1) $$J=\frac{\Delta W}{A•\Delta t},$$

where ∆*W* (Kg) is the weight of permeation, *A* (m^2^) is the total effective membrane area, and Δ*t* (h) is the operation time.

The normalized/relative flux (%) before and after fouling was calculated by Equation (2):

(2) $$J_{N}=\frac{J_{i}}{J_{0}}\times 100\%,$$

where *J*_0_ (Kg·m^−2^·h^−1^) is the initial flux, and *J_i_* (Kg·m^−2^·h^−1^) is the instantaneous flux during the filtration of real industrial sample, which could cause flux decline due to fouling.

The rejection (*R*) of solute was calculated by Equation (3):

(3) $$R=\frac{C_{f0}-C_{pt}}{C_{f0}},$$

where *C_f0_* (mg/L) is the total dissolved solids (TDS) in the original feed, and *C_pt_* (mg/L) is the TDS concentration in the permeate water collected at time t.

## 3. Results and Discussion

### 3.1. Membrane Characterization

**Figure 2:**
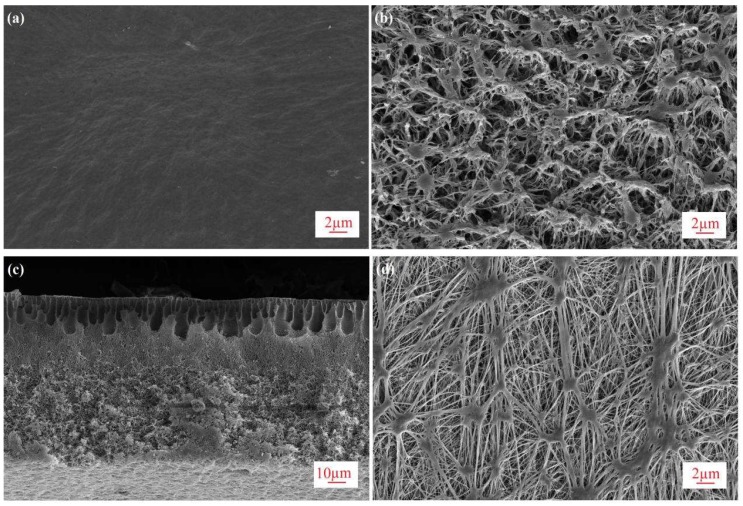
SEM images of membranes (**a**) top surface of virgin PVDF membrane (5000×); (**b**) bottom surface of virgin PVDF membrane (5000×); (**c**) cross section of virgin PVDF membrane (1000×); (**d**) top surface of virgin PTFE membrane (5000×).

The membranes used in this study are asymmetric PVDF and commercial PTFE membranes. The characteristics of the PVDF membrane are given in Table 1; the characteristics of the commercial PTFE membrane were provided by the manufacturer and the relevant literature [33,34]. The PVDF membrane has a much smaller mean pore size (r_m_) of 34 nm than that of PTFE (450 nm) [33,34], leading to a much higher liquid entry pressure of water (LEP_w_) of 3.5 bar, indicating excellent anti-wetting properties. Aside from the high porosity (ε) of 86%, which is similar to that of the PTFE membrane [33,34], the total membrane thickness (δ) is as thin as 95 μm, which is indicative of high permeability. SEM images of both membranes are shown in Figure 2. In Figure 2a,d, it was found that the asymmetric PVDF membrane exhibits a dense and smooth top surface, which is significantly different from the rough fibrous structure of the commercial PTFE membrane. The asymmetric structure of the PVDF membrane with an ultra-thin skin top surface, finger-like pores, and a bicontinuous network beneath the skin are observed in the cross section in Figure 2c. 

### 3.2. Effects of Operating Parameters in DCMD

#### 3.2.1. Effect of Flow Rate

**Figure 3:**
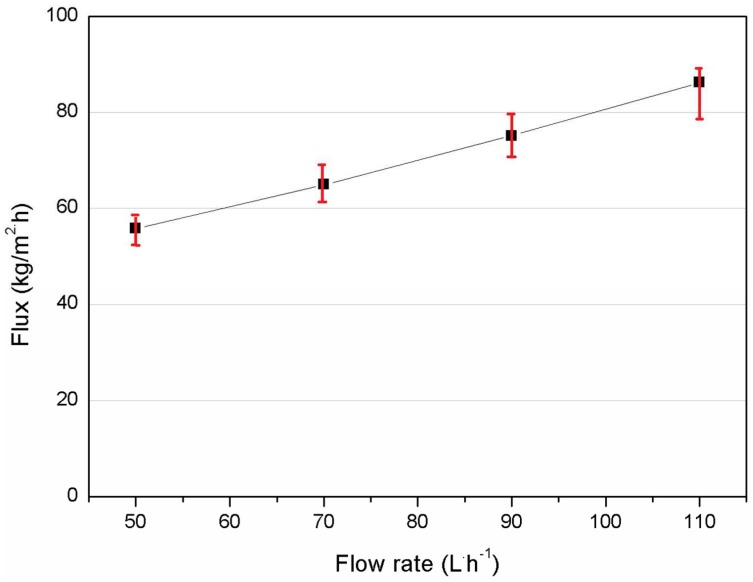
Effect of flow rate on permeation flux of asymmetric PVDF membrane in DCMD (*C*_f_ = 3.5 wt % NaCl, T_f_ = 70 °C, T_p_ = 16 °C, Q_f_ = Q_p_).

Figure 3 shows the relationship between permeation flux and the flow rates of feed and permeate, where the flow rate of both sides were kept the same. The membrane flux increases as the flow rate increases from 50 to 110 L/h (linear velocity: 0.28–0.61 m/s). This is because the increase of flow rate helps reduce the thickness of the liquid boundary layer adjacent to the membrane surface, which alleviates the effect of concentration and temperature polarization, resulting in enhanced mass and heat transfer coefficients [38]. Thus, it improves the process driving force and subsequently permeation flux. Similar investigations on flow rate have been reported [12,39]. Therefore, the highest flow rate of 110 L/h within the testing range was selected for the following tests. It is also noted that during the above DCMD experiments the salt rejection was stable at 99.99% to ensure membrane integrity. 

#### 3.2.2. Effect of Membrane Orientation

**Figure 4:**
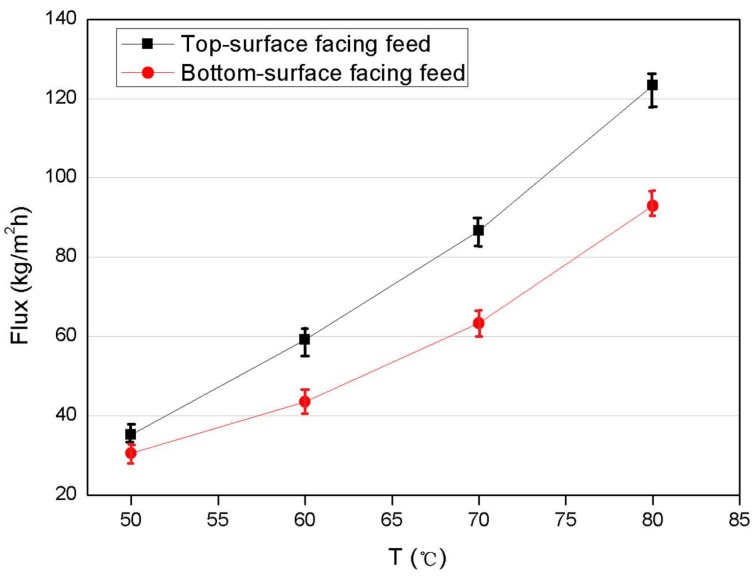
Effect of membrane orientation on permeation flux of asymmetric PVDF in DCMD at varying feed temperature (*C*_f_ = 3.5 wt % NaCl, T_p_ = 16 °C, Q_f_ = Q_p_ = 110 L·h^−1^).

The effect of membrane orientation was investigated at varying feed temperatures from 50 to 80 °C, i.e., with the feed solution facing the top or bottom surface of the membrane. The results are presented in Figure 4. Compared to the bottom-surface-facing feed mode, the top-surface facing the feed solution produces at least 25% higher flux. For example, the flux of the top-surface facing the feed mode showed up to 123 kg·m^−2^·h^−1^ at 80 °C. This is due to the different pore structure of the two surfaces of the asymmetric PVDF membrane fabricated by the NTIPS method, producing an ultra-thin, dense and smooth top surface exhibiting no macropores that is potentially smaller than the mean free path (<0.11 μm) of the water molecules and thus will likely follow the Knudsen diffusion mechanism in the classic MD mass transfer model [40,41]; the highly porous and rough bottom surface of the membrane exhibits much larger pores and hence may fall into the regime of combined Knudsen/molecular diffusion [39,42]. Thus, MD flux involving the Knudsen mechanism is considered higher than that of the combined Knudsen/molecular diffusion mechanism, as reported in the literature [32,43]. Hence, the orientation of top surface facing the feed was used in subsequent investigations. It is noted that the salt rejection was stable at 99.99% in the above DCMD tests for both orientations.

#### 3.2.3. Effect of Feed Salinity

**Figure 5:**
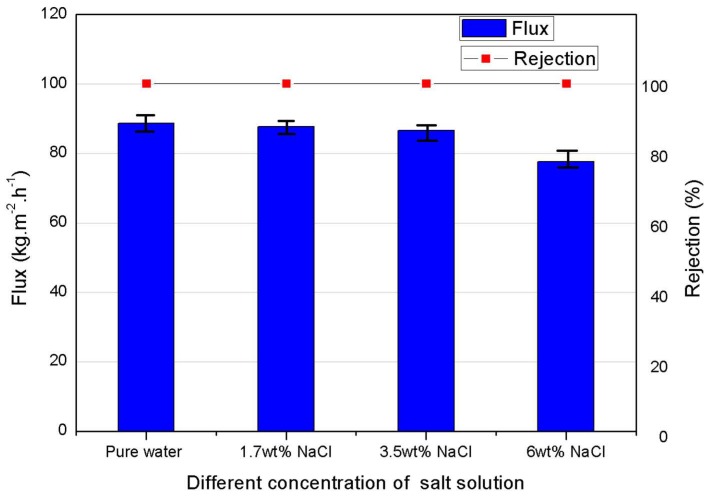
Effects of different salt concentrations of feed (T_f_ = 70 °C, T_p_ = 16 °C, δ = 95 µm, Q_f_ = Q_p_ = 110 L·h^−1^).

Four synthetic solutions with varying salinity from 0 to 60 g/L were tested in DCMD with the asymmetric PVDF membranes. The influence of salinity on the permeation flux is presented in Figure 5. It was found that the permeate flux decreased slightly by 12%, i.e., from 88.6 to 77.6 kg·m^−2^·h^−1^, as the salt concentration increased from 0 to 6 wt %. This can be explained by the reduction of vapor pressure and water activity coefficient of the feed when increasing the solute concentration [9], which leads to decreased driving force for vapor transport in MD. However, within a given salinity range, the concentration polarization effect was not known to significantly affect the flux. Overall, the membrane performance was only slightly influenced by the salt concentration of the feed, up to the salinity level of the real industrial sample to be investigated in this work. 

### 3.3. Concentration of Real Organic Fertilizer by DCMD

In this section, with the synthetic seawater testing as a benchmark, the concentration of real organic fertilizer was measured using both the as-prepared PVDF and commercial PTFE membranes. 

#### 3.3.1. Permeation Flux of Organic Fertilizer as Feed

**Figure 6:**
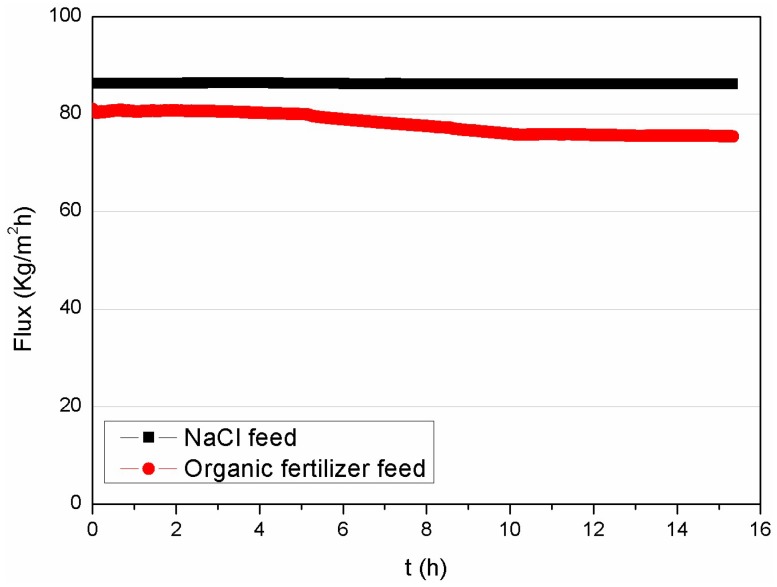
Continuous DCMD runs of organic fertilizer and NaCl feed using asymmetric PVDF membrane (T_f_ = 70 °C, T_p_ = 16 °C, Q_f_ = Q_p_ = 110 L·h^−1^).

**Figure 7:**
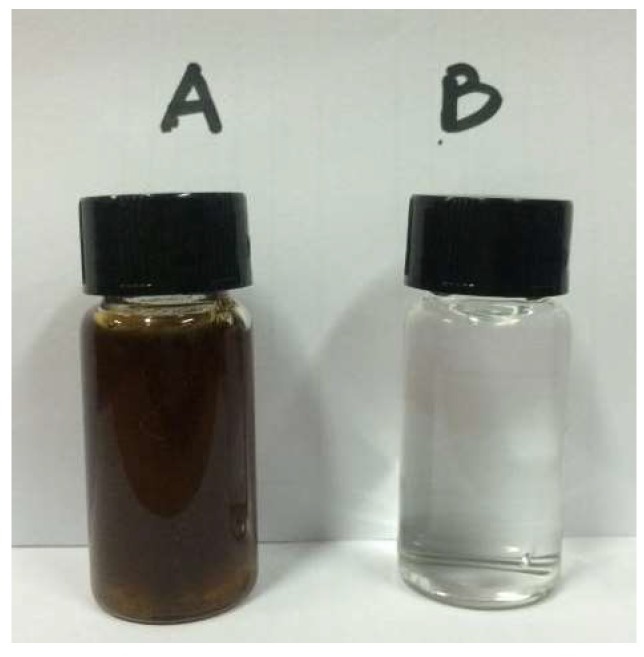
Images of original organic fertilizer sample (**A**) and MD permeate sample (**B**).

The PVDF membrane performance was evaluated by testing both the NaCl solution (3.5 wt % at the beginning) and an organic fertilizer in 15-h continuous DCMD runs in batch mode to concentrate the fertilizer. The concentration results are presented in terms of permeation flux, as illustrated in Figure 6. The initial flux of the organic fertilizer feed was around 80 kg·m^−2^·h^−1^, which is similar to that of the synthetic seawater, i.e., 86 kg·m^−2^·h^−1^. Although a minor decrease in the flux was observed for the industrial sample after 15 h of operation, it still remained around 74 kg·m^−2^·h^−1^, where 2.6x concentration has been achieved to obtain a fertilizer of 7.8% organic matter, i.e., 63.4% water was removed from the fertilizer sample containing approximately 3% organic nutrients. It is noted that the TDS rejection of the membrane was stable at 99.99% for both feed solutions. Figure 7 shows a comparison of the original organic fertilizer sample (A) and the permeate (B). The feed solution is turbid and dark brown, which is in contrast to the transparent permeate solution. 

#### 3.3.2. Membrane Fouling and Surface Inspection

**Figure 8:**
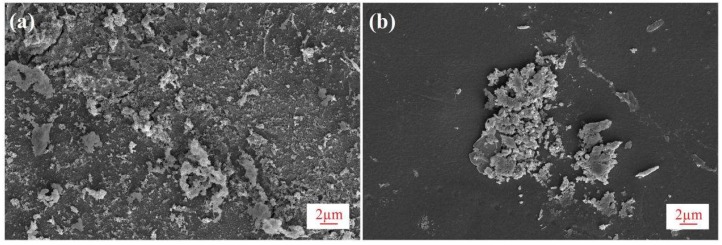
SEM images of PVDF membrane after 15-h continuous DCMD experiments (**a**) fouled membrane after 15 h operation (5000×); (**b**) cleaned membrane rinsed with pure water (5000×).

**Figure 9:**
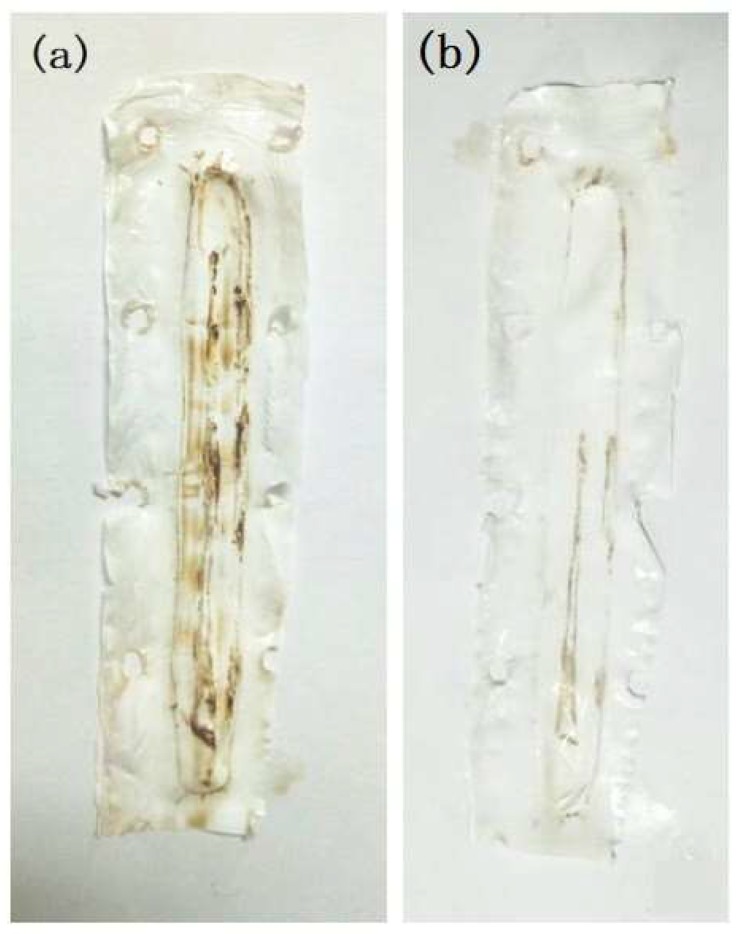
Pictures of as-prepared PVDF membrane (**a**) fouled membrane after fertilizer testing and (**b**) cleaned membrane rinsed with pure water.

As a result of the 15-h continuous operation of the organic fertilizer, flux decline was observed. This may be due to the reduction in vapor pressure of the feed solution and hence the transmembrane driving force as concentration increased. Also, the build-up of the fouling layer on the surface of the PVDF membrane could cause further flux decline, as evidenced by the surface inspection by SEM in Figure 8 and Figure 9. To reveal the fouling behavior of the PVDF membrane, surface inspection was carried out. Figure 8 shows the SEM images of the fouled membrane (a) and the cleaned membrane rinsed with pure water (b). Correspondingly, Figure 9 shows pictures of the fouled (A) and cleaned membrane (B). As shown in Figure 8a and Figure 9a, the entire surface of the PVDF membrane was covered by a layer of amorphous deposition, which has the same dark brown color as the fertilizer feed; the fouling layer was almost completely removed through clean water rinsing, as shown in Figure 8b and Figure 9b. The easy cleaning of the membrane after 15 h of running may be attributed to the unique dense and smooth top surface structure of the asymmetric PVDF membrane. 

#### 3.3.3. Comparison of the Anti-Fouling Performance of Different Membranes

**Figure 10:**
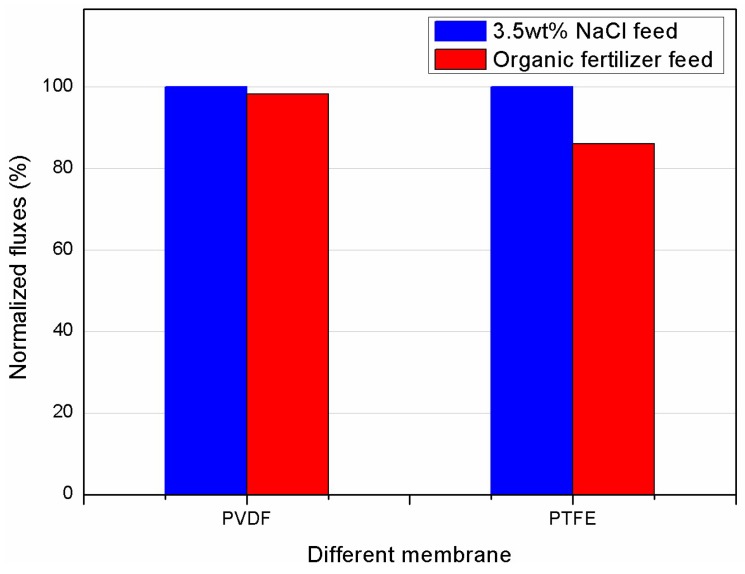
Comparison of initial normalized fluxes of as-prepared asymmetric PVDF and commercial PTFE membrane with synthetic and organic fertilizer (T_f_ = 70 °C, T_p_ = 16 °C, Q_f_ = Q_p_ = 110 L·h^−1^).

The anti-fouling property of the asymmetric PVDF membrane has been further investigated in comparison to the commercial PTFE membrane. The normalized fluxes (Equation (2)) were used to evaluate the fouling tendency associated with performance deterioration of both membranes. Figure 10 shows the normalized flux of both membranes, where both a synthetic 3.5 wt % salt solution and organic fertilizer feed were tested in the initial 1-h experiments. Compared to 14% flux decrease of the PTFE membrane, the PVDF membrane showed only a minor flux decline of 1.8%, indicating a more sustainable performance in treating challenging feed solutions.

**Figure 11:**
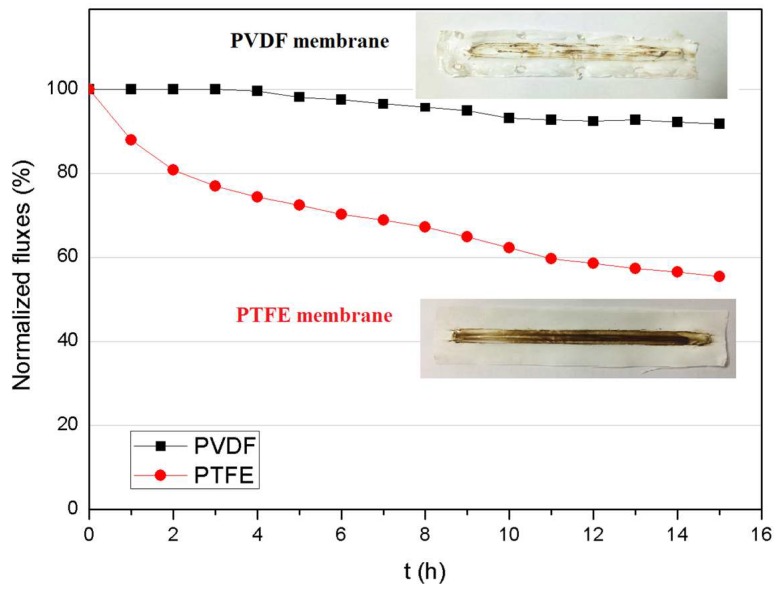
Comparison of normalized flux of asymmetric PVDF and commercial PTFE membranes with organic fertilizer tested in 15-h continuous MD runs (*C*_f_ = organic fertilizer feed, T_f_ = 70 °C, T_p_ = 16 °C, Q_f_ = Q_p_ = 110 L·h^−1^).

The comparison of normalized fluxes of the asymmetric PVDF and commercial PTFE membrane was further investigated with an organic fertilizer feed in a 15-h continuous operation. The results are shown in Figure 11, in which the normalized flux of the PVDF membrane exhibits a very slow and minor decrease of 8% and remains relatively constant after a 15-h continuous operation. In contrast, a rapid decrease was observed with the PTFE membrane, resulting in 56% flux decline after 15 h. This could be due to the quick build-up of the fouling layer on the surface of the PTFE membrane, which exhibits a rough and fibrous surface structure, as indicated in Figure 2. Pictures of the fouled membranes after the 15-h operation are given in Figure 11: the deposit on the smooth surface of the PVDF membrane (top picture) was minor and relatively loose, while the cake layer on the PTFE membrane was dense.

## 4. Conclusions

Membrane distillation is an emerging technology for solute concentration and value recovery from aqueous streams. As an alternative to the conventional MSF process, the potential of MD to be applied in the concentration of a semi-product of organic fertilizer was evaluated with a unique asymmetric PVDF membrane prepared by the unconventional NTIPS method. The MD process’s stability was examined in terms of the membrane integrity and fouling tendency associated with flux loss and membrane cleaning. Investigations revealed that the asymmetric PVDF membrane exhibited superior permeability up to 86 kg·m^−2^·h^−1^ at 70 °C. Consistent with the literature data, the membrane flux increased with increasing flow rate and decreasing solution salinity. Interestingly, the selection of membrane orientation, i.e., top-surface- or bottom-surface-facing feed mode, was proven to be important in determining the membrane permeability. As a result, the top-surface-facing feed mode was chosen due to the smaller pore size contributed by the Knudsen diffusion mechanism of mass transport. Furthermore, compared to the commercial PTFE membrane, the asymmetric PVDF membrane showed superior sustainability in permeability and fouling propensity, maintaining more than 92% membrane flux after a 15-h continuous operation. The flux was easily recovered by simple water rinsing. As a result, a 2.6x concentration factor was achieved in one MD run. Thus, the potential to achieve a much higher concentration factor is feasible with MD due to the ease of flux recovery and the excellent anti-fouling and anti-wetting properties of the as-developed PVDF membrane. 

## Figures and Tables

**Table 1:** Characterization of asymmetric PVDF membrane and PTFE membrane.

Membrane Type	Porosity (ε, %)	LEP_w_ (Bar)	Mean Pore Size (r_m_, nm)	Total Thickness (δ, µm)	Contact Angle (θ, °)
Asymmetric PVDF	86 ± 1	3.5 ± 0.1	34 ± 3	95 ± 5	85 ± 3
Commercial PTFE	92.5 ± 0.5	0.8 ± 0.05	450 ± 50	36 ± 1 (PTFE layer)	140 ± 2.5
